# Bacterial sexually transmitted infections among men who have sex with men and transgender women using oral pre-exposure prophylaxis in Latin America (ImPrEP): a secondary analysis of a prospective, open-label, multicentre study

**DOI:** 10.1016/S2352-3018(24)00211-X

**Published:** 2024-09-05

**Authors:** Mayara Secco Torres Silva, Thiago Silva Torres, Carolina Coutinho, Ronaldo Ismério Moreira, Iuri da Costa Leite, Marcelo Cunha, Pedro Henrique Amparo da Costa Leite, Carlos F Cáceres, Hamid Vega-Ramírez, Kelika A Konda, Juan Guanira, José Valdez Madruga, Sandra Wagner Cardoso, Marcos Benedetti, Maria Cristina Pimenta, Brenda Hoagland, Beatriz Grinsztejn, Valdilea Gonçalves Veloso, ImPrEP Study Group, Marcus Vinicius Lacerda, José Valdez Madruga, Alessandro Farias, Josué N Lima, Ronaldo Zonta, Lilian Lauria, J. David Urbaez-Brito, Polyana d'Albuquerque, Claudio Palombo, Paulo Ricardo Alencastro, Raquel Keiko de Luca Ito, Júlio Moreira, João L. Benedetti, Fabio V. Maria, Paula M. Luz, Lucilene Freitas, Kim Geraldo, Monica Derrico, Sandro Nazer, Tania Kristic, Renato Girade, Renato Lima, Antônio R. Carvalho, Carla Rocha, Pedro Leite, Marcio Lessa, Marilia Santini-Oliveira, Daniel R.B. Bezerra, Cleo de Oliveira Souza, Jacinto Corrêa, Marcelo Alves, Carolina Souza, Camilla Portugal, Mônica dos Santos Valões, Gabriel Lima Mota, Joyce Alves Gomes, Cynthia Ferreira Lima Falcão, Fernanda Falcão Riberson, Luciano Melo, Talita Andrade Oliveira, Agnaldo Moreira Oliveira Júnior, Bruna Fonseca, Leonor Henriette Lannoy, Ludymilla Anderson Santiago Carlos, João Paulo Cunha, Sonia Maria de Alencastro Coracini, Thiago Oliveira Rodrigues, Emília Regina Scharf Mettrau, Kelly Vieira Meira, Heder Tavares, Ana Paula Nunes Viveiros Valeiras, Taiane Miyake Alves de Carvalho Rocha, Alex Amorim, Patrícia Sabadini, Luiz Gustavo Córdoba, Caio Gusmão, Erika Faustino, Julia Soares da Silva Hansen, Agatha Mirian Cunha, Neuza Uchiyama Nishimura, Jaime Eduardo Flygare Razo Prereira Santos, Aline Barnabé Cano, Willyam Magnum Telles Dias, Magô Tonhon, Tania Regina Rezende, Alex Gomes, Eloá dos Santos Rodrigues, Maria das Dores Aires Carneiro, Alexandre Castilho, Mariana Carvalho, Sergio Bautista-Aredondo, Heleen Vermandere, Steven Diaz, Dulce Diaz-Sosa, Centli Guillen-Diaz-Barriga, Rebeca Robles, Maria Elena Medina-Mora, Marcela González, Ivonne Huerta Icelo, Araczy Martinez Davalos, José Gomez Castro, Luis Obed Ocampo Valdez, Fernanda Ramírez Barajas, Verónica Ruiz González, Galileo Vargas Guadarrama, Israel Macías, Jehovani Tena Sánchez, Juan Pablo Osuna Noriega, H. Rodrigo Moheno M., Jorge M. Bernal Ramírez, Víctor Dante Galicia Juarez, Gerardo Vizcaíno, Francisco Javier Arjona, Cesar Vidal Osco Tamayo, Hector Javier Salvatierra Flores, Yovanna Margot Cabrera Santa Cruz, Ricardo Martín Moreno Aguayo, Gino Calvo, Silver Vargas, Oliver Elorreaga, Ximena Gutierrez, Fernando Olivos, Damaris Caviedes, Daniella Adriazola, Eduardo Juárez, Gabriela Mariño, Jazmin Qquellon, Francesca Vasquez, Jean Pierre Jiron, Sonia Flores, Karen Campos

**Affiliations:** a Instituto Nacional de Infectologia Evandro Chagas, Fundação Oswaldo Cruz (INI-Fiocruz), Rio de Janeiro, Brazil; b Escola Nacional de Saúde Pública Sérgio Arouca, Fundação Oswaldo Cruz (ENSP-Fiocruz), Rio de Janeiro, Brazil; c Universidad Peruana Cayetano Heredia, Centro de Investigaciones Interdisciplinarias en Salud, Sexualidad, y SIDA, Lima, Peru; d Instituto Nacional de Psiquiatria Ramón de la Fuente Muñiz, Mexico City, Mexico; e Keck School of Medicine, University of Southern California, Los Angeles, CA, USA; f Centro de Referência e Treinamento em DST/AIDS—CRT-SP, São Paulo, Brazil

## Abstract

**Background:**

The global burden of sexually transmitted infections (STIs) poses a challenge in the context of HIV pre-exposure prophylaxis (PrEP) programmes. We aimed to explore factors associated with prevalent, incident, and recurrent STIs in men who have sex with men (MSM) and transgender women on PrEP in Brazil, Mexico, and Peru.

**Methods:**

ImPrEP was a prospective, single-arm, open-label, multicentre study that enrolled MSM and transgender women in the context of the public health systems of Brazil (14 sites), Mexico (four sites), and Peru (ten sites) between February, 2018, and June, 2021. Eligibility criteria followed regional PrEP guidelines at the study start, including participants aged 18 years and older, not living with HIV, and reporting at least one of the following in the previous 6 months: condomless anal sex (CAS), anal sex with partner(s) living with HIV, any bacterial STI, or transactional sex. Eligible participants were screened and enrolled on the same day to receive daily oral PrEP (tenofovir disoproxil fumarate 300 mg and emtricitabine 200 mg). We assessed three outcomes: prevalent bacterial STIs, incident bacterial STIs, and recurrent bacterial STIs. Testing occurred at baseline and quarterly for syphilis, anorectal chlamydia, and anorectal gonorrhoea. Behavioural data were collected at baseline and quarterly. The study was registered with the Brazilian Registry of Clinical Trials, U1111-1217-6021.

**Findings:**

Among all 9509 participants included in the ImPrEP study (3928 [41·3%] in Brazil, 3288 [34·6%] in Mexico, and 2293 [24·1%] in Peru), 8525 (89·7%) had available STI results at baseline and were included in the prevalent STI analysis, and 7558 (79·5%) had available STI results during follow-up and were included in the incident and recurrent STI analyses. 2184 (25·6%) of 8525 participants had any bacterial STI at baseline. STI incidence during follow-up was 31·7 cases per 100 person-years (95% CI 30·7–32·7), with the highest rate for anorectal chlamydia (11·6 cases per 100 person-years, 95% CI 11·0–12·2), followed by syphilis (10·5 cases per 100 person-years, 9·9–11·1) and anorectal gonorrhoea (9·7 cases per 100 person-years, 9·2–10·3). Although only 2391 (31·6%) of 7558 participants had at least one STI during follow-up, 915 (12·1%) participants had recurrent diagnoses, representing 2328 (61·2%) of 3804 incident STI diagnoses. Characteristics associated with prevalent, incident, and recurrent STIs included younger age, multiple sex partners, receptive CAS, substance use, and previous STI diagnoses at baseline (incident or recurrent only).

**Interpretation:**

Our findings underscore the nuanced dynamics of STI transmission among MSM and transgender women across Latin America, highlighting an urgent need for tailored interventions to mitigate STI burden effectively, especially among the most susceptible individuals.

**Funding:**

Unitaid, WHO, and ministries of health (Brazil, Mexico, and Peru).

**Translations:**

For the Portuguese and Spanish translations of the abstract see Supplementary Materials section.

## Introduction

According to WHO, 374 million adults globally acquired new infections of chlamydia, gonorrhoea, syphilis, or thrichomoniasis in 2020.^1^ Active STIs increase the odds of acquisition and transmission of additional infections including HIV, and are a point of interest in the context of combined prevention promoted throughout the roll-out of pre-exposure prophylaxis (PrEP) programmes.^2^ Men who have sex with men (MSM) and transgender women are disproportionally affected by HIV and other STIs.^3^, ^4^, ^5^, ^6^ Recent outbreaks of pathogens transmitted by sexual contact, such as mpox, enteric bacteria, and hepatitis A, and increasing emergence of resistant sexually transmitted pathogens and enterobacteria have been reported among MSM, especially in Europe.^7^, ^8^

A meta-analysis of PrEP trials that mainly enrolled MSM from high-income locations after 2015 reported a pooled prevalence of any bacterial STI (syphilis, gonorrhoea, or chlamydia) of 23·9% (95% CI 18·6–29·6), with an incidence of 72·2 (95% CI 60·5–86·2) cases per 100 person years.^3^ STI surveillance within PrEP programmes provides an unprecedented opportunity to inform stakeholders in planning implementation of comprehensive sexual health services to MSM and transgender women. These populations are heavily affected by structural and societal factors, including stigma and discrimination,^9^ which in Latin America add to barriers to implement adequate diagnosis methods and prompt treatment.^10^ In 2019, STI incidence in the region reached 12 955·71 cases per 100 000 person-years.^1^ A meta-analysis exploring data on syphilis among MSM globally found the highest prevalence in the Latin America region (10·6%, 95% CI 8·5–12·9).^6^

**Research in context**

**Evidence before this study**

We searched PubMed between database inception and April 30, 2024, using the keywords “PrEP” or “pre-exposure prophylaxis”, combined with “sexually transmitted infections” (including “syphilis”, “gonorrhea”, and “chlamydia”), along with geographical qualifiers (“Latin America”, “Brazil”, “Mexico”, and “Peru”). There were no restrictions on publication date or language, resulting in the identification of 38 relevant manuscripts. Our review focused on literature concerning the incidence of sexually transmitted infections (STIs) during pre-exposure prophylaxis (PrEP) use among gay, bisexual, and other cisgender men who have sex with men (MSM), as well as transgender women, in Latin America. Despite the availability of PrEP through the public health systems of Brazil (since December, 2017), Mexico (since mid-2021), and Peru (since August, 2023), its scale-up remains restricted in the region. We found reports on STI incidence among PrEP users from implementation studies in Brazil and clinical trials such as iPrEX and HPTN083. However, evidence regarding incident STIs in Latin America remains scarce, with most published data primarily focusing on prevalence rates. Notably, none of these studies delved into factors associated with incident and recurrent STIs among PrEP users. Existing data indicate a substantial burden of STIs among MSM and PrEP users in Latin America, particularly syphilis.

**Added value of this study**

The Implementation PrEP (ImPrEP) study was the largest PrEP implementation study in Latin America, evaluating the feasibility of same-day oral PrEP in the context of the public health systems of Brazil, Mexico, and Peru. The ImPrEP study reported data on prevalent and incident STIs for 8525 participants, drawing attention to the high prevalence and incidence of bacterial STIs among study participants, including syphilis, anorectal chlamydia, and anorectal gonorrhoea. Age younger than 30 years, self-identification as a non-White race, low educational background, high number of sexual partners, and receptive condomless anal sex (CAS) were associated with baseline prevalence of any bacterial STI. During study follow-up, younger age, non-White race, previous HIV post-exposure prophylaxis use, high number of sexual partners, receptive CAS, use of poppers, and STI diagnosis at enrolment were associated with incident and recurrent bacterial STIs. Regional differences can also be involved in STI transmission dynamics among PrEP users. We found STI incident diagnoses in about 30% of participants and recurrent STI diagnoses in 12% of participants. Participants diagnosed with any STI showed high adherence to PrEP, and reported multiple vulnerabilities related to sociodemographic and behavioural characteristics before starting PrEP.

**Implications of all the available evidence**

Our findings underscore the substantial burden of STIs in Latin America, reinforcing the role of PrEP services as crucial venues for both diagnosis and timely treatment of these conditions. Furthermore, our findings emphasise the importance of adopting a combined prevention approach. Despite the observed high incidence of STIs, our data suggest that individuals diagnosed with an STI had various vulnerabilities before starting PrEP. Engagement in combination prevention services facilitated regular sexual health assessments, increased testing, awareness, and access to appropriate treatment. Consequently, the expansion of PrEP programmes might be a crucial strategy in curbing both HIV and STI transmission. Moreover, the identification of factors associated with incident and recurrent STIs among PrEP users holds promise for enhancing our understanding of transmission dynamics, which is particularly pertinent given the emergence of new biomedical prevention strategies for bacterial STIs.

Few longitudinal studies have analysed incident STIs among PrEP users in Latin America.^10^, ^11^, ^12^ Cross-sectional analyses enrolling MSM and transgender women on PrEP in cities across Brazil and Ecuador indicated a high burden of bacterial STIs, including syphilis, chlamydia, and gonorrhoea.^13^, ^14^, ^15^, ^16^ Analyses of incident STIs reveal particularly high syphilis rates, especially in Brazil.^13^, ^17^, ^18^, ^19^, ^20^, ^21^ None of these studies reported on patterns of STI incidence or factors associated with incident or recurrent STIs in the region. Implementation PrEP (ImPrEP), the largest implementation study to evaluate same-day oral PrEP initiation for MSM and transgender women in Latin America, provides a valuable opportunity to further understand STI transmission dynamics in Latin America. In this secondary analysis, we aimed to explore the factors associated with prevalent, incident, and recurrent STIs during the ImPrEP study.

## Methods

### Study design and participants

ImPrEP was a prospective, single-arm, open-label, multicentre study that enrolled MSM and transgender women in the context of the public health systems in Brazil (14 sites), Mexico (four sites), and Peru (ten sites) between February, 2018, and June, 2021.^22^ Eligibility criteria followed regional PrEP guidelines at the study start, included individuals aged 18 years and older, not living with HIV, and reporting at least one of the following in the previous 6 months: condomless anal sex (CAS), anal sex with partner(s) living with HIV, any bacterial STI, or transactional sex. Gender was self-reported and participants could choose between cisgender man, cisgender woman, transgender man, transgender woman, and travesti. We excluded participants who self-identified as cisgender women or transgender men.

The study was approved by institutional review boards at Instituto Nacional de Infectologia Evandro Chagas, INI-Fiocruz (Rio de Janeiro, Brazil; CAAE:79259517.5.1001.5262), National Institute of Public Health (Mexico City, Mexico; CI-1515), and Universidad Peruana Cayetano Heredia (Lima, Peru; 100740). Ethical approval was also obtained from the WHO Research Ethics Review Committee and local institutional review boards at each Brazilian site. All study participants provided written informed consent before enrolment in Portuguese (Brazil) or Spanish (Mexico and Peru). The study was registered with the Brazilian Registry of Clinical Trials, U1111-1217-6021.

### Procedures

Eligible participants were screened and enrolled on the same day to receive daily oral PrEP (tenofovir disoproxil fumarate 300 mg and emtricitabine 200 mg). Follow-up visits were scheduled at week 4 and quarterly (every 3 months) thereafter during the period of study, and at each visit participants were prescribed PrEP, behavioural information was collected, and further examinations were scheduled accordingly. For behavioural information, we used a recall period of 3 months for both the baseline and follow-up visits, except for CAS (recall period of 6 months at the baseline visit, and 3 months for all follow-up visits). Syphilis testing comprised a rapid *Treponema pallidum* test, followed by non-treponemal tests if positive (Brazil and Mexico: venereal disease research laboratory [VDRL] test; Peru: rapid plasma reagin [RPR] test), whereas molecular detection of chlamydia or gonorrhoea was done in samples obtained from self-collected anal swabs (Brazil: Abbott Real Time platform and CT/NG Amplification Reagent Kit [Abbott Molecular; Des Plains, IL, USA]; Mexico: STD Direct Flow Chip [Máster Diagnóstica; Granada, Spain]; Peru: Aptima Combo 2 assay [Hologic; Sandiego, CA, USA]).

Gender was dichotomised into cisgender men and transgender women. We described age at baseline both as numerical (median [IQR]) and categorical ranges (18–24 years, 25–30 years, >30 years). Self-reported race was categorised as non-White (Asian, Black, Indigenous, or mixed-race [Pardo or Mestizo]) versus White. Schooling was stratified into primary, secondary, or post-secondary education. Post-exposure prophylaxis use 12 months before baseline was dichotomised (yes *vs* no). Sexual behaviour was retrieved by asking the number of sex partners (0–1, 2–3, ≥4), CAS (yes *vs* no), CAS with partner with HIV (yes, no, do not know), transactional sex (sex in exchange for money, drugs, gifts, favours, etc), and substance use (stimulant drugs [ecstasy, lysergic acid diethylamide, gamma-hydroxybutyrate, cocaine or derivatives, or ketamine], poppers [inhaled alkyl nitrates], or binge drinking). Adequate PrEP adherence was defined as having a medication possession ratio of at least 0·6, equivalent to at least four tenofovir disoproxil fumarate and emtricitabine pills per week.^22^

All STI tests were done at baseline (visit 0) and every 3 months (from visit 2 onwards). Anorectal chlamydia and gonorrhoea was tested annually (visits 0, 6, and 10) until June, 2020, and at every follow-up visit (visit 2, every 3 months) thereafter. Interim visits were done if a participant presented with any adverse events, including STI-related symptoms, and treatment was provided as per the local standard of care. Additional samples for chlamydia and gonorrhoea testing could be collected based on clinical judgement and local availability, but only anorectal samples provided by the study were analysed, with results recorded at the most recent visit. This decision was based on the restricted access to molecular diagnosis in the study countries and the high rates of asymptomatic chlamydia and gonorrhoea at extragenital sites among MSM and transgender women, particularly anorectal.

### Outcomes

We assessed three outcomes in this analysis: prevalent bacterial STIs, incident bacterial STIs, and recurrent bacterial STIs. Prevalent bacterial STI was defined as the presence of active syphilis (titres ≥1:8 and a positive microhaemagglutination assay for *T pallidum*), anorectal chlamydia, rectal gonorrhoea, or all of these, at baseline. Incident bacterial STI was defined as the occurrence of the first incident episode of syphilis or anorectal chlamydia or anorectal gonorrhoea anytime during follow-up. For chlamydia and gonorrhoea, an incident diagnosis was considered if a positive result followed a previous negative result. Incident syphilis was defined as a new positive treponemal test detected during any visit, if negative at baseline. For participants who were already positive for syphilis at baseline, incident diagnoses were identified by a four-times titre increase in the VDRL or RPR result compared with the previous test. In instances where a participant had a previous syphilis diagnosis during the ImPrEP follow-up, we considered incident cases only if the participant showed a serological response to treatment, characterised by a four-times titre increase in the VDRL or RPR result compared with the visit when the diagnosis was initially made. A recurrent bacterial STI was defined as either a second incident STI diagnosis following a previous STI, or as the simultaneous diagnosis of at least two different pathogens (syphilis, anorectal chlamydia, or anorectal gonorrhoea) at the time of the initial infection.

Assessment of safety and adverse events during the ImPrEP study has been described elsewhere.^22^

### Statistical analysis

Baseline sociodemographic and behavioural characteristics of all participants included in the analyses were described. We included all ImPrEP participants who took any bacterial STI test (syphilis or anorectal chlamydia or gonorrhoea) at enrolment in prevalence analysis, whereas only those who attended at least one follow-up visit and had been tested at least once after baseline were considered for incidence or recurrence analysis. We calculated incidence rates with 95% CIs for any bacterial STI and each STI separately, and person-years under follow-up considering time between baseline and the last visit. Bacterial STIs diagnosed at baseline were not included in the numerator of STI incidence rates. We calculated the proportion of any incident bacterial STI (syphilis, anorectal chlamydia, or anorectal gonorrhoea) per participant, and the number of times when any incident bacterial STI was diagnosed after baseline per participant.

We calculated the overall STI and STI-specific incidence rates using a Poisson model with the number of person-years of follow-up as the offset. To identify factors associated with prevalent STIs, we used a log-binomial model, interpreting the results as prevalence ratios. For factors related to incident and recurrent STIs, we used a Cox regression model with time-dependent variables, where each participant was followed up from enrolment until event occurrence or censoring. As the hazards in the Cox models with time-dependent variables were no longer proportional, we investigated whether using averaged hazard ratios (HRs) was reasonable for interpreting coefficients related to the explanatory variables over the follow-up period by using the graphical diagnosis of scaled Schoenfeld residuals. Multicollinearity was evaluated using the variance inflation factor, with a threshold of 2·5. The analyses were done in two stages. Initially, we evaluated each risk factor while controlling solely for country. Factors statistically significant at the 10% level were then included in the final models, where the interpretation was made in terms of the association measures and their respective 95% CIs. Given the limited advantages of imputing outcome variable values, we did not impute missing values of the STI variable at baseline for the prevalent STI analysis. Since baseline STI was an explanatory variable in the incident and recurrent STI analyses, missing values were addressed through multiple imputation. This process used variables measured at baseline and behavioural time-dependent variables measured at each quarterly visit, generating 20 imputed datasets via the predictive mean matching method, considering the longitudinal data structure, with the 2lonly.norm command available in the Mice package of the R software.

Statistical analyses were done with R (version 4.2.2).

### Role of the funding source

The funders of the study had no role in study design, data collection, data analysis, data interpretation, or writing of the report.

## Results

Among all 9509 participants included in the ImPrEP study, 8525 (89·7%) had available STI results at baseline and were included in the prevalent STI analysis (3640 [92·7%] in Brazil, 2671 [81·2%] in Mexico, and 2214 [96·6%] in Peru). Participants were mostly cisgender men, aged younger than 30 years, non-White, and had post-secondary education (table 1). 7006 (82·2%) of 8525 participants reported no previous post-exposure prophylaxis use, 5519 (65·7%) reported four or more sex partners, 5586 (65·5%) reported receptive CAS, and 4469 (52·4%) reported sex with a partner of unknown HIV status. Transactional sex, binge drinking, stimulant use, and use of poppers were also reported. Comparison of participants included versus not included in the prevalent STI analysis per country are provided in appendix 3 (pp 1–2).

**Table 1:** Sociodemographic and behavioural characteristics of ImPrEP participants

		**Included in prevalent STI analysis**			**Included in incident and reccurent STI analyses**		
		Yes (n=8525)	No (n=984)	p value*	Yes (n=7558)	No (n=1951)	p value*
Country		..	..	<0·0001	..	..	<0·0001
	Brazil	3640 (42·7%)	288 (29·3%)	..	3447 (45·6%)	481 (24·7%)	..
	Mexico	2671 (31·3%)	617 (62·7%)	..	2486 (32·9%)	802 (41·1%)	..
	Peru	2214 (26·0%)	79 (8·0%)	..	1625 (21·5%)	668 (34·2%)	..
Gender		..	..	<0·0001	..	..	<0·0001
	Cisgender man	8011 (94·0%)	955 (97·1%)		7211 (95·4%)	1755 (90·0%)	..
	Transgender woman	514 (6·0%)	29 (2·9%)		347 (4·6%)	196 (10·0%)	..
Age, years		..	..	..	..	..	..
	Median (IQR)	29 (24–35)	29 (25–35)	0·054	27 (23–32)	29 (25–35)	<0·0001
	18–24	2249 (26·4%)	232 (23·6%)	0·16	1831 (24·2%)	650 (33·3%)	<0·0001
	25–30	2736 (32·1%)	331 (33·6%)	..	2399 (31·7%)	668 (34·2%)	..
	>30	3540 (41·5%)	421 (42·8%)	..	3328 (44·0%)	633 (32·4%)	..
Race		..	..	0·0002	..	..	<0·0001
	White	2339 (27·4%)	215 (21·9%)	..	2201 (29·1%)	353 (18·1%)	..
	Non-White	6186 (72·6%)	769 (78·2%)	..	5357 (70·9%)	1598 (81·9%)	..
Education		..	..	<0·0001	..	..	<0·0001
	Primary	102 (1·2%)	11 (1·1%)	..	76 (1·0%)	37 (1·9%)	..
	Secondary	1551 (18·2%)	111 (11·3%)	..	1191 (15·8%)	471 (24·1%)	..
	Post-secondary	6872 (80·6%)	862 (87·6%)	..	6291 (83·2%)	1443 (74·0%)	..
Main reason to attend the service		..	..	<0·0001	..	..	<0·0001
	Seeking pre-exposure prophylaxis	7414 (87·0%)	934 (94·9%)	..	6771 (89·6%)	1577 (80·8%)	..
	Other	1111 (13·0%)	50 (5·1%)	..	787 (10·4%)	374 (19·2%)	..
Previous post-exposure prophylaxis use		..	..	0·85	..	..	<0·0001
	Yes	1519 (17·8%)	173 (17·6%)	..	1484 (19·6%)	208 (10·7%)	
	No	7006 (82·2%)	811 (82·4%)	..	6074 (80·4%)	1743 (89·3%)	
Number of sex partners†		..	..	0·0009	..	..	0·0001
	0–1	1254 (14·7%)	144 (14·6%)	..	1124 (14·9%)	274 (14·0%)	..
	2–3	1752 (20·6%)	154 (15·7%)	..	1456 (19·3%)	450 (23·1%)	..
	≥4	5519 (64·7%)	686 (69·7%)	..	4978 (65·9%)	1227 (62·9%)	..
	Median (IQR)	7 (3–17)	5 (2–15)	<0·0001	5 (2–15)	6 (3–15)	0·056
Receptive CAS‡		..	..	0·18	..	..	0·063
	Yes	5586 (65·5%)	666 (67·7%)	..	5004 (66·2%)	1248 (64·0%)	..
	No	2939 (34·5%)	318 (32·3)	..	2554 (33·8%)	703 (36·0%)	..
CAS with partner(s) with HIV‡		..	..	<0·0001	..	..	<0·0001
	Yes	1634 (19·2%)	258 (26·2%)	..	1593 (21·1%)	299 (15·3%)	..
	No	2421 (28·4%)	219 (22·3%)	..	2107 (27·9%)	533 (27·3%)	..
	Do not know	4469 (52·4%)	507 (51·5%)	..	3858 (51·0%)	1118 (57·3%)	..
Transactional sex		..	..	0·28	..	..	<0·0001
	Yes	1485 (17·4%)	158 (16·1%)	..	1168 (15·4%)	475 (24·4%)	..
	No	7039 (82·6%)	826 (83·9%)	..	6390 (84·6%)	1475 (75·6%)	..
Binge drinking†		..	..	0·0017	..	..	0·027
	Yes	5651 (66·3%)	603 (61·3%)	..	4930 (65·2%)	1324 (67·9%)	..
	No	2873 (33·7%)	381 (38·7%)	..	2628 (34·8%)	626 (32·1%)	..
Stimulant use†		..	..	0·088	..	..	0·86
	Yes	1543 (18·1%)	200 (20·3%)	..	1388 (18·4%)	355 (18·2%)	..
	No	6982 (81·9%)	784 (79·7%)	..	6170 (81·6%)	1596 (81·8%)	..
Use of poppers†		..	..	<0·0001	..	..	0·21
	Yes	1631 (19·1%)	308 (31·3%)	..	1561 (20·6%)	378 (19·4%)	..
	No	6894 (80·9%)	676 (68·7%)	..	5997 (79·4%)	1573 (80·6%)	..
Any bacterial STI at enrolment		..	..	..	..	..	0·89
	Yes	NA	NA	NA	1733 (22·9%)	451 (23·1%)	..
	No	NA	NA	NA	5023 (66·5%)	1318 (67·6%)	..
	Not tested	NA	NA	NA	802 (10·6%)	182 (9·3%)	..

Data are n (%), unless otherwise indicated. CAS=condomless anal sex. NA=not applicable. STI=sexually transmitted infection.

* χ^2^ test or Kruskal-Wallis test.

† In the last 3 months.

‡ In the last 6 months.

2184 (25·6%) of 8525 participants had any bacterial STI at baseline, mainly anorectal chlamydia (1008 [11·8%] participants), followed by syphilis (817 [9·6%] participants) and anorectal gonorrhoea (793 [9·3%] participants). Prevalent STI diagnosis was higher among participants of younger age (18–30 years), of non-White race, with primary or secondary education, attending the service not seeking PrEP, reporting more than one sex partner, and reporting receptive CAS (table 2). Compared with Brazil, prevalent STIs were lower among participants from Mexico; the prevalence in Brazil did not significantly differ from Peru.

**Table 2:** Factors associated with prevalent, incident, and recurrent STI diagnoses among ImPrEP participants

	**Prevalent STI diagnosis**				**Incident STI diagnosis**				**Recurrent STI diagnosis**			
	Univariable analyses		Multivariable analysis		Univariable analyses		Multivariable analysis		Univariable analyses		Multivariable analysis	
	Prevalence rate (95% CI)	p value	Adjusted prevalence rate (95% CI)	p value	HR (95% CI)	p value	Adjusted HR (95% CI)	p value	HR (95% CI)	p value	Adjusted HR (95% CI)	p value
**Country**												
Brazil	1 (ref)	..	1 (ref)	..	1 (ref)	..	1 (ref)	..	1 (ref)	..	1 (ref)	
Mexico	0·98 (0·90–1·07)	0·65	0·85 (0·76–0·94)	0·0021	1·46 (1·34–1·60)	<0·0001	1·54 (1·36–1·75)	<0·0001	1·85 (1·61–2·14)	<0·0001	2·01 (1·65–2·46)	<0·0001
Peru	1·12 (1·02–1·22)	0·014	0·96 (0·86–1·06)	0·39	0·82 (0·72–0·92)	<0·0001	0·72 (0·63–0·82)	<0·0001	0·66 (0·53–0·82)	0·0002	0·54 (0·43–0·68)	<0·0001
**Gender**												
Cisgender man	1 (ref)	..	1 (ref)	..	1 (ref)	..	1 (ref)	..	1 (ref)	..	NA	NA
Transgender woman	1·44 (1·27–1·62)	<0·0001	1·15 (1·00–1·33)	0·054	1·27 (1·05–1·53)	0·015	0·95 (0·77–1·18)	0·63	1·06 (0·75–1·50)	0·72	NA	NA
**Age, years**												
18–24	1·32 (1·21–1·44)	<0·0001	1·30 (1·19–1·42)	<0·0001	1·30 (1·17–1·44)	<0·0001	1·22 (1·10–1·35)	0·0002	1·53 (1·30–1·81)	<0·0001	1·46 (1·23–1·72)	<0·0001
25–30	1·16 (1·07–1·27)	0·0007	1·17 (1·07–1·27)	0·0005	1·14 (1·04–1·26)	0·0047	1·10 (1·00–1·20)	0·073	1·22 (1·04–1·42)	0·012	1·16 (0·99–1·35)	0·062
>30	1 (ref)	..	1 (ref)	..	1 (ref)	..	1 (ref)	..	1 (ref)	..	1 (ref)	..
**Race**												
White	1 (ref)	..	1 (ref)	..	1 (ref)	..	1 (ref)	..	1 (ref)	..	1 (ref)	..
Non-White	1·18 (1·07–1·29)	0·0006	1·16 (1·06–1·27)	0·0015	1·16 (1·05–1·27)	0·0023	1·13 (1·03–1·24)	0·017	1·20 (1·03–1·40)	0·018	1·15 (0·99–1·35)	0·069
**Education**												
Primary	1·60 (1·25–2·04)	0·0002	1·36 (1·07–1·74)	0·013	0·99 (0·64–1·52)	0·97	0·98 (0·63–1·52)	0·93	1·24 (0·64–2·40)	0·52	1·20 (0·62–2·34)	0·60
Secondary	1·22 (1·12–1·34)	<0·0001	1·12 (1·02–1·23)	0·023	1·16 (1·04–1·30)	0·011	1·06 (0·94–1·20)	0·34	1·19 (0·99–1·44)	0·069	1·04 (0·85–1·26)	0·70
More than secondary	1 (ref)	..	1 (ref)	..	1 (ref)	..	1 (ref)	..	1 (ref)	..	1 (ref)	..
**Main reason to attend the service**												
Seeking pre-exposure prophylaxis	1 (ref)	..	1 (ref)	..	1 (ref)	..	NA	NA	1 (ref)	..	NA	NA
Other	1·23 (1·10–1·37)	0·0002	1·17 (1·05–1·30)	0·0049	1·06 (0·91–1·24)	0·48	NA	NA	1·16 (0·89–1·51)	0·28	NA	NA
**Post-exposure prophylaxis use**												
Yes	1·00 (0·90–1·10)	0·99	NA	NA	1·16 (1·05–1·28)	0·0030	1·13 (1·02–1·24)	0·014	1·13 (0·97–1·32)	0·12	NA	NA
No	1 (ref)	..	NA	NA	1 (ref)	..	1 (ref)	..	1 (ref)	..	NA	NA
Number of sex partners*												
0–1	1 (ref)	..	1 (ref)	..	1 (ref)	..	1 (ref)	..	1 (ref)	..	1 (ref)	..
2–3	1·23 (1·07–1·41)	0·0036	1·19 (1·03–1·37)	0·015	1·53 1·33–1·75)	<0·0001	1·47 (1·29–1·69)	<0·0001	1·95 (1·54–2·48)	<0·0001	1·84 (1·45–2·34)	<0·0001
≥4	1·40 (1·24–1·58)	<0·0001	1·30 (1·14–1·48)	<0·0001	1·94 (1·73–2·17)	<0·0001	1·75 (1·55–1·98)	<0·0001	2·63 (2·14–3·23)	<0·0001	2·28 (1·84–2·82)	<0·0001
**Receptive CAS**†												
Yes	1·34 (1·24–1·46)	<0·0001	1·26 (1·16–1·37)	<0·0001	1·63 (1·48–1·80)	<0·0001	1·47 (1·33–1·62)	<0·0001	1·91 (1·63–2·24)	<0·0001	1·66 (1·41–1·95)	<0·0001
No	1 (ref)	..	1 (ref)	..	1 (ref)	..	1 (ref)	..	1 (ref)	..	1 (ref)	..
**CAS with partner(s) with HIV**†												
Yes	1·02 (0·91–1·14)	0·73	1·04 (0·93–1·17)	0·45	1·00 (0·89–1·12)	0·99	1·03 (0·91–1·16)	0·64	0·97 (0·80–1·18)	0·77	1·01 (0·83–1·22)	0·95
No	1 (ref)	..	1 (ref)	..	1 (ref)	..	1 (ref)	..	1 (ref)	..	1 (ref)	..
Do not know	1·18 (1·08–1·28)	0·0002	1·07 (0·98–1·17)	0·12	1·11 (1·00–1·22)	0·039	1·00 (0·91–1·10)	1·00	1·19 (1·01–1·39)	0·035	1·04 (0·89–1·22)	0·60
**Transactional sex**												
Yes	1·28 (1·17–1·39)	<0·0001	1·06 (0·96–1·17)	0·28	1·30 (1·17–1·45)	<0·0001	1·05 (0·93–1·19)	0·38	1·29 (1·08–1·54)	0·0048	0·93 (0·77–1·13)	0·47
No	1 (ref)	..	1 (ref)	..	1 (ref)	..	1 (ref)	..	1 (ref)	..	1 (ref)	..
Binge drinking*												
Yes	1·04 (0·96–1·13)	0·30	NA	NA	1·06 (0·98–1·15)	0·16	NA	NA	1·09 (0·96–1·25)	0·19	NA	NA
No	1 (ref)	..	NA	NA	1 (ref)	..	NA	NA	1 (ref)	..	NA	NA
Stimulant use*												
Yes	1·17 (1·06–1·28)	0·0010	1·07 (0·97–1·18)	0·16	1·25 (1·12–1·39)	<0·0001	1·05 (0·93–1·17)	0·43	1·36 (1·15–1·61)	0·0004	1·10 (0·92–1·31)	0·29
No	1 (ref)	..	1 (ref)	..	1 (ref)	..	1 (ref)	..	1 (ref)	..	1 (ref)	..
Use of poppers*												
Yes	1·15 (1·04–1·28)	0·0080	1·12 (1·00–1·25)	0·049	1·37 (1·22–1·54)	<0·0001	1·18 (1·04–1·33)	0·0081	1·51 (1·26–1·81)	<0·0001	1·25 (1·04–1·52)	0·021
No	1 (ref)	..	1 (ref)	..	1 (ref)	..	1 (ref)	..	1 (ref)	..	1 (ref)	..
**Bacterial STI at baseline**												
Yes	NA	NA	NA	NA	1·78 (1·62–1·95)	<0·0001	1·66 (1·51–1·82)	<0·0001	2·17 (1·88–2·50)	<0·0001	1·99 (1·72–2·30)	<0·0001
No	NA	NA	NA	NA	1 (ref)	..	1 (ref)	..	1 (ref)	..	1 (ref)	..

CAS=condomless anal sex. HR=hazard ratio. NA=not applicable. STI=sexually transmitted infection.

* In the last 3 months.

† In the last 6 months.

For the incident and recurrent analyses, we included 7558 (79·5%) of 9509 ImPrEP participants who had at least one STI test during follow-up visits (Brazil: 3447 [45·6%] participants; Mexico: 2486 [32·9%] participants; Peru: 1625 [21·5%] participants). 1796 (23·8%) of 7558 participants were lost to follow-up, including 69 individuals who acquired HIV before any STI was detected. Overall, 7211 (95·4%) of 7558 participants were cisgender MSM and 347 (4·6%) were travesti or transgender women, and 4230 (56·0%) were aged 30 years or younger. Those not included in the incident and recurrent STI analyses were more frequently from Mexico and Peru compared with Brazil, transgender women versus cisgender men, aged 18–24 years versus older than 24 years, and non-White versus White, and more likely to have secondary education or lower versus post-secondary education (table 1).

Participants were followed up for a total of 11 959·9 person-years after enrolment. Overall, the follow-up period ranged from 0·2 to 3·3 years, with a median follow-up of 1·6 years (IQR 0·8–2·3). Participants without STIs had a shorter median follow-up compared with those diagnosed with any bacterial STI (1·4 years, IQR 0·7–2·1 *vs* 1·9 years, 1·3–2·5; p<0·0001). 2160 (28·6%) of 7558 participants were followed up for less than 1 year. The median number of STI tests per person-year during the follow-up period was eight (IQR 4–12), comprising four (2–6) for syphilis and two (1–3) for anorectal chlamydia or gonorrhoea. 3804 STIs were diagnosed (1257 syphilis cases, 1159 anorectal gonorrhoea cases, and 1388 anorectal chlamydia cases). The incidence of any bacterial STI during follow-up was 31·7 cases per 100 person-years (95% CI 30·7–32·7), with the highest rate for anorectal chlamydia (11·6 cases per 100 person-years, 95% CI 11·0–12·2), followed by syphilis (10·5 cases per 100 person-years, 9·9–11·1) and anorectal gonorrhoea (9·7 cases per 100 person-years, 9·2–10·3). The number of bacterial STIs diagnosed during follow-up per participant ranged from zero to nine (median 0, IQR 0–1). 5167 (68·4%) of 7558 participants had no bacterial STI diagnosed during follow-up, and 2391 (31·6%) of 7558 participants accounted for all incident diagnoses. 1476 (19·5%) of 7558 participants had one incident bacterial STI, 599 (7·9%) participants had two incident bacterial STIs, 200 (2·6%) participants had three incident bacterial STIs, 74 (1·0%) had four incident bacterial STIs, and 42 (0·6%) had five or more incident bacterial STIs (table 3; figure). 915 (12·1%) participants had recurrent STI diagnoses, representing 2328 (61·2%) of 3804 STI incident diagnoses. Adequate PrEP adherence was high when measured at the visit when the first incident STI occurred (1958 [81·9%] of 2391 participants).

**Table 3:** Incident bacterial STIs diagnosed per participant during the ImPrEP study

	**Participants with any incident bacterial STI (n=7558)**	**Incident bacterial STI diagnoses (n=3804)**
0	5167 (68·4%)	0
1	1476 (19·5%)	1476 (38·8%)
2	599 (7·9%)	1198 (31·5%)
3	200 (2·6%)	600 (15·8%)
4	74 (1·0%)	296 (7·8%)
≥5	42 (0·6%)	234 (6·2%)

Data are n (%). STI=sexually transmitted infection.

**Figure:**
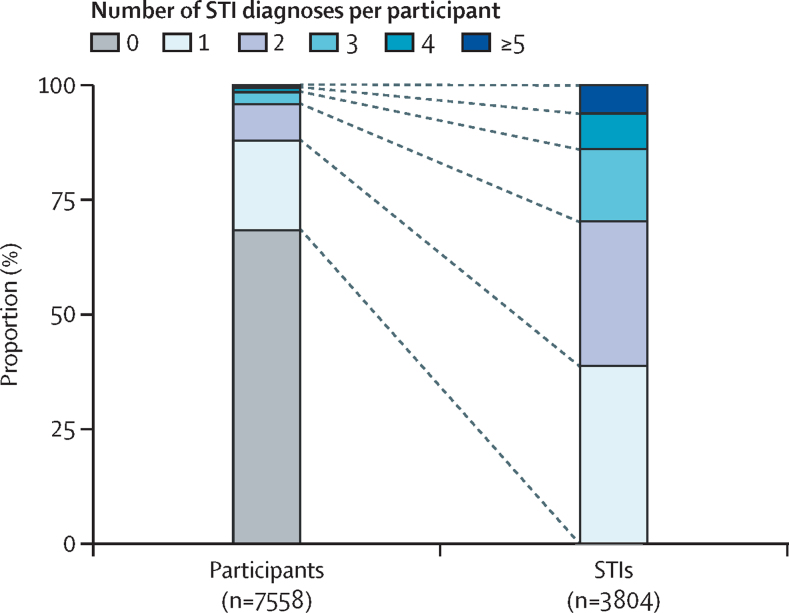
Number of bacterial STIs diagnosed during ImPrEP study follow-up according to the proportion of diagnoses per participant STI=sexually transmitted infection.

Incident bacterial STIs were higher among participants from Mexico, those aged 18–24 years, those who self-identified as non-White, those who reported previous post-exposure prophylaxis use at baseline, those who reported multiple sex partners since the last visit, those who reported receptive CAS, those who reported use of poppers, and those with previous STI diagnosis at baseline (table 2).

Recurrent bacterial STIs were higher among those aged 18–24 years, those who reported multiple sex partners since the last visit, those who reported use of poppers, and those with previous STI diagnosis at baseline (table 2). In relation to specific countries, compared with Brazil, recurrent bacterial STIs were higher among participants from Mexico but lower among those from Peru (table 2).

The characteristics of participants included in incident and recurrent STI analyses were similar across countries, with some differential losses among participants with increased vulnerability to HIV or other STIs (appendix 3 pp 3–4). Factors associated with incident and recurrent STIs per country are described in appendix 3 (pp 5–10).

## Discussion

In this large cohort of MSM and transgender women using oral PrEP in Latin America, we found a high burden of STIs, especially among those aged 18–30 years, those with secondary education or lower, and those self-identifying as non-White. Substance use, previous post-exposure prophylaxis prescription, transactional sex, and self-reported behavioural characteristics, including high frequency of CAS and a higher number of sex partners, were associated with increased probabilities of being diagnosed with any bacterial STI at baseline, before PrEP initiation, and during follow-up, after PrEP initiation. International cohorts also reported similar factors associated with incident and recurrent bacterial STI diagnoses after PrEP initiation, including younger age, higher number of sexual partners, CAS, and substance use.^23^, ^24^, ^25^, ^26^, ^27^

Our data indicate a high concentration of bacterial STI diagnoses among a small proportion of individuals, as more than 70% of participants had no bacterial STI diagnosis during follow-up. 12% of participants accounted for 61% of all incident bacterial STIs diagnosed during the study follow-up, and recurrent STI diagnoses were more frequent among those of younger age (18–24 years), with a higher number of sex partners, and reporting poppers use. Moreover, being diagnosed with any bacterial STI at baseline posed an increased risk of incident and recurrent STIs during study follow-up. These findings might help to identify individuals in Latin America who could benefit most from biomedical technologies aiming to prevent bacterial STIs, such as the use of doxycycline as post-exposure prophylaxis.^28^, ^29^, ^30^ Additionally, tailored interventions considering comprehensive sexual behaviour counselling, strengthening public policies on sex education in schools, and harm reduction strategies to mitigate substance use should be considered while implementing STI prevention programmes in the region.

The high rates of incident and recurrent bacterial STIs are in line with the regional landscape, since Latin America faces epidemics of HIV and STIs, especially syphilis, which disproportionally burden MSM and transgender women.^4^, ^6^, ^9^ Compared with STI incidence in PrEP cohorts from high-income countries, for example Australia (91·9 cases per 100 person-years), Canada and France (75·0 cases per 100 person-years), and the USA (48·3 cases per 100 person-years), the incidence rate of any bacterial STI was lower among ImPrEP participants (31·7 cases per 100 person-years).^23^, ^24^, ^25^ Different regional patterns have been reported in terms of each individual bacterial STI. The Australian cohort estimated a higher incidence of anorectal chlamydia (34·3 cases per 100 person-years) and anorectal gonorrhoea (22·6 cases per 100 person-years) compared with syphilis (8 cases per 100 person-years). Anorectal chlamydia (13 cases per 200 person-years) and anorectal gonorrhoea (15 cases per 100 person-years) rates in France and Canada and the USA (12·2 cases per 100 person-years for chlamydia and 7·4 cases per 100 person-years for gonorrhoea) were similar to ImPrEP estimates (11·6 cases per 100 person-years for anorectal chlamydia and 9·7 cases per 100 person-years for anorectal gonorrhoea). Syphilis incidence rates in ImPrEP were similar to France and Canada (13·0 cases per 100 person-years *vs* 10·5 cases per 100 person-years in ImPrEP), but higher than those reported in the USA (8·7 cases per 100 person-years) and Australia (8·0 cases per 100 person-years).^23^, ^24^, ^25^

Additionally, the HPTN083 study, which evaluated long-acting injectable PrEP with cabotegravir among 4566 MSM and transgender women, of whom 1964 (43%) were from Latin America, also found high STI incidence (50·7 cases per 100 person-years), which was highest in Africa (75 cases per 100 person-years) and Latin America (63·9 cases per 100 person-years). Syphilis and rectal chlamydia had equal incidence rates (16·7 cases per 100 person-years), followed by rectal gonorrhoea (11 cases per 100 person-years), with few cases of urogenital chlamydia (4·5 cases per 100 person-years) or gonorrhoea (2·4 cases per 100 person-years). Similar with our data, about 60% of participants had no STI diagnosis during the study, and almost 80% of all STI diagnoses were found in only 25% of participants.^27^ Therefore, our findings are in line with these STI incidence data in a trial including a high proportion of participants from Latin America.^27^

Our study has several limitations. We did not collect multisite swab samples for chlamydia or gonorrhoea and tested for these infections less frequently compared with syphilis, which might have led to underestimation of chlamydia and gonorrhoea rates. We did not systematically conduct tests of cure nor systematically collect information on STI treatment. We used laboratory cure criteria as a proxy for STI resolution by comparing previous and incident bacterial STI, potentially underestimating new diagnoses. Higher proportions of participants reporting STI-related vulnerabilities (eg, younger age, non-White race, and lower education) were not included in incidence analysis, which could have underestimated incidence rates in the ImPrEP study. The HR for assessing the association between risk factors and STI incidence could be affected by selection bias due to unequal weighting of early and late HRs. Although almost a quarter of participants were lost to follow-up, this bias is mitigated by the relatively short median follow-up period of 1·6 years and similar follow-up times regardless of STI diagnosis. Self-reported data on sexual behaviour and substance use might be subject to recall bias, and our analyses could be affected by unmeasured confounders. Previous data suggest that participants from Mexico and Peru, transgender women, those identifying as non-White, those with lower education, those with fewer sex partners, those with no history of transactional sex, and those younger than 30 years have increased odds of early loss to follow-up.^22^ These observations might explain the lower risk of recurrent STIs in Peru and the increased risk of incident and recurrent STIs in Mexico. Strategies to improve retention on PrEP and routine sexual health assessments are crucial for effective HIV and STI prevention.

Our cohort is highly representative of MSM and transgender women using oral PrEP in Latin America and brings important data on prevalent, incident, and recurrent STIs and their associated risk factors. Overtly, data from ImPrEP contribute to further describing the landscape of bacterial STIs among MSM and transgender women in Latin America, providing data on molecular diagnosis of STIs among these key populations in the region.

In conclusion, our results add to a growing body of evidence that monitors data on STIs in Latin America, outlining the importance of age, sexual behaviour, and substance use in further understanding the dynamics of STI transmission in the region. Our findings suggest an already high vulnerability to STIs at PrEP initiation, and incidence of recurrent STIs among a specifc group of individuals as the main drivers of the high burden of bacterial STIs among people on PrEP. Therefore, the intertwining of person-centred approaches to adequately identify those more vulnerable to bacterial STIs, comprehensive sexual health assessments in PrEP programmes, and intersectional actions to tackle insufficient sex education and the burden of mental health conditions and substance use are crucial to curb the HIV and STI syndemic in Latin America.

### Contributors

### Data sharing

A complete de-identified dataset sufficient to reproduce this analysis will be made available upon request to the corresponding author, following approval of a concept sheet summarising the analyses to be done.

## Declaration of interests

We declare no competing interests.

## Supplementary Materials

Portuguese translation of the abstract

Spanish translation of the abstract
