# Supplementary material for: Bacterial sexually transmitted infections among men who have sex with men and transgender women using oral pre-exposure prophylaxis in Latin America (ImPrEP): a secondary analysis of a prospective, open-label, multicentre study
Source: Lancet HIV. 2024 Sep 5;11(10):e670–9. doi: 10.1016/S2352-3018(24)00211-X (PMC11442320; doi:10.1016/S2352-3018(24)00211-X)
Supplement: Portuguese translation of the abstract [file mmc1.pdf]

THE LANCET
HIV

Supplementary appendix 1

This translation in Portuguese was submitted by the authors and we reproduce it as supplied. It has not been peer reviewed. *The Lancet*'s editorial processes have only been applied to the original in English, which should serve as reference for this manuscript.

Esta tradução em português foi submetida pelos autores e nós não fizemos quaisquer alterações. Esta versão não foi revista por pares. O processo editorial do The Lancet só foi aplicado à versão original em inglês, que deve servir como referência para este artigo.

Supplement to: Torres Silva MS, Torres TS, Coutinho C, et al. Bacterial sexually transmitted infections among men who have sex with men and transgender women using oral pre-exposure prophylaxis in Latin America (ImPrEP): a secondary analysis of a prospective, open-label, multicentre study. *Lancet HIV* 2024; published online Sept 4. https://doi.org/10.1016/S2352-3018(24)00211-X.

Resumo [300/300]

Introdução

As infecções sexualmente transmissíveis(IST) são uma preocupação crescente nos programas de profilaxia pré-exposição ao HIV(PrEP). Este estudo analisa os fatores associados a IST prevalentes, incidentes e recorrentes entre homens que fazem sexo com homens(HSH), travestis e mulheres trans em uso de PrEP na América Latina.

Métodos

O ImPrEP foi um estudo prospectivo, aberto e multicêntrico conduzido no Brasil, México e Peru entre fevereiro/2018 e junho/2021, incluindo HSH, travestis e mulheres trans com idade≥18 anos, negativos para HIV, que relataram sexo anal sem preservativo, sexo anal com parceria vivendo com HIV, diagnóstico IST e/ou sexo transacional nos últimos 6 meses. Todos receberam PrEP oral diária (300 mg tenofovir-disoproxil-fumarato/200 mg emtricitabina). Foram analisadas IST prevalentes, incidentes e recorrentes, com testes realizados na inclusão e trimestralmente para sífilis e anualmente para clamídia e gonorreia anorretal. ID do estudo: UTN U1111-1217-6021.

Resultados

Dentre os 9509 participantes, 8525(89,7%) tinham resultados de IST disponíveis na inclusão, com prevalência de IST na inclusão de 25%(n=2184). 7558(79,5%) tinham resultados de IST durante o seguimento, com incidência de 31,7/100 pessoas-ano(IC 95% 30,7–32,7), sendo maior para clamídia anorretal(11,6/100 pessoas-ano;IC 95% 11,0–12,2), seguida de sífilis(10,5/100 pessoas-ano;IC 95% 9,9–11,1) e gonorreia anorretal (9,7 por 100 pessoas-ano, 9,2–10,3). Apenas 2391(31,6%) dos 7558 participantes foram diagnosticados com pelo menos uma IST durante o seguimento, e 915(12,1%) participantes tiveram diagnósticos recorrentes, representando 2328(61,2%) dos 3804 diagnósticos de IST incidentes. Fatores associados a IST prevalentes, incidentes e recorrentes incluíram idade mais jovem, múltiplos parceiros sexuais, sexo anal receptivo sem preservativo, uso de substâncias e diagnósticos prévios de IST na linha de base (apenas incidentes ou recorrentes).

Discussão

Nossos resultados destacam a carga desproporcional de IST entre minorias sexuais e de gênero na América Latina, destacando a necessidade de intervenções preventivas e abordagens interseccionais para mitigar a carga de IST na região.
