# Supplementary material for: Bacterial sexually transmitted infections among men who have sex with men and transgender women using oral pre-exposure prophylaxis in Latin America (ImPrEP): a secondary analysis of a prospective, open-label, multicentre study
Source: Lancet HIV. 2024 Sep 5;11(10):e670–9. doi: 10.1016/S2352-3018(24)00211-X (PMC11442320; doi:10.1016/S2352-3018(24)00211-X)
Supplement: Spanish translation of the abstract [file mmc2.pdf]

THE LANCET
HIV

## Supplementary appendix 2

This translation in Spanish was submitted by the authors and we reproduce it as supplied. It has not been peer reviewed. *The Lancet*'s editorial processes have only been applied to the original in English, which should serve as reference for this manuscript.

Los autores nos proporcionaron esta traducción al español y la reproducimos tal como nos fue entregada. No la hemos revisado. Los procesos editoriales de *The Lancet* se han aplicado únicamente al original en inglés, que debe servir de referencia para este manuscrito.

Supplement to: Torres Silva MS, Torres TS, Coutinho C, et al. Bacterial sexually transmitted infections among men who have sex with men and transgender women using oral pre-exposure prophylaxis in Latin America (ImPrEP): a secondary analysis of a prospective, open-label, multicentre study. *Lancet HIV* 2024; published online Sept 4. https://doi.org/10.1016/S2352-3018(24)00211-X.

**Resumen** [300/300]

**Antecedentes**

Las infecciones de transmisión sexual(ITS) son una preocupación creciente en los programas de profilaxis preexposición al VIH(PrEP). Este estudio analiza los factores asociados a las ITS prevalentes, incidentes y recurrentes entre hombres que tienen sexo con hombres(HSH), travestis y mujeres trans(TMT) que usan PrEP en América Latina.

**Métodos**

ImPrEP fue un estudio prospectivo, abierto y multicéntrico realizado en Brasil, México y Perú entre febrero/2018 y junio/2021, e incluyó HSH y TMT de ≥18 años, negativos al VIH y que tuvieron sexo anal sin preservativo, sexo anal con pareja viviendo con VIH, diagnóstico previo de ITS y/o sexo transaccional(últimos 6 meses). Todos recibieron PrEP oral diaria(tenofovir-disoproxil-fumarato[300mg]/emtricitabina[200mg]). Se analizó la prevalencia, incidencia y recurrencia de ITS con las pruebas realizadas a la inclusión, cada trimestre(sífilis) y anualmente(clamidia y gonorrea anorrectal). ID del estudio: UTN U1111-1217-6021.

**Resultados**

De los 9,509 participantes, hubo 8,525(89.7%) resultados disponibles de ITS en la inclusión, con una prevalencia de 25%(n=2,184). Hubo 7,558 (79.5%) resultados de ITS disponibles durante el seguimiento, con una incidencia de 31.7/100 personas-año (IC95%:30.7–32.7), siendo mayor para clamidia anorrectal(11.6/100 personas-año; IC95%:11.0–12.2), seguida de sífilis (10.5/100 personas-año; IC95%:9.9–11.1) y gonorrea anorrectal (9.7/100 personas-año; IC95%:9.2–10.3). Solo 2,391 (31,6%) de los 7,558 participantes fueron diagnosticados con al menos una ITS durante el seguimiento y 915 (12.1%) tuvieron diagnósticos recurrentes, representando 2,328 (61.2%) de los 3,804 diagnósticos de ITS incidentes. Los factores asociados con ITS prevalentes, incidentes y recurrentes fueron menor edad, múltiples parejas sexuales, sexo anal receptivo sin preservativo, uso de sustancias y diagnósticos previos de ITS al momento de la inclusión (solo para incidentes o recurrentes).

**Discusión**

Nuestros resultados destacan la carga desproporcionada de ITS entre minorías sexuales y de género en América Latina, subrayando la necesidad de intervenciones preventivas y abordajes interseccionales para mitigar la carga de ITS en la región.
