# Supplementary material for: Bacterial sexually transmitted infections among men who have sex with men and transgender women using oral pre-exposure prophylaxis in Latin America (ImPrEP): a secondary analysis of a prospective, open-label, multicentre study
Source: Lancet HIV. 2024 Sep 5;11(10):e670–9. doi: 10.1016/S2352-3018(24)00211-X (PMC11442320; doi:10.1016/S2352-3018(24)00211-X)
Supplement: Supplementary appendix 3 [file mmc3.pdf]

THE LANCET
HIV

Supplementary appendix 3

This appendix formed part of the original submission and has been peer reviewed. We post it as supplied by the authors.

Supplement to: Torres Silva MS, Torres TS, Coutinho C, et al. Bacterial sexually transmitted infections among men who have sex with men and transgender women using oral pre-exposure prophylaxis in Latin America (ImPrEP): a secondary analysis of a prospective, open-label, multicentre study. *Lancet HIV* 2024; published online Sept 4. https://doi.org/10.1016/S2352-3018(24)00211-X.

Supplementary Material

	Page
Supplementary Table 1. Baseline characteristics of participants according to inclusion in the prevalent STI analysis (per country)	1
Supplementary Table 2. Baseline characteristics of participants according to inclusion in the incidence and recurrent STI analyses (per country)	3
Supplementary Table 3. Factors associated with incident and recurrent bacterial STI diagnoses among ImPrEP participants from Brazil	5
Supplementary Table 4. Factors associated with incident and recurrent bacterial STI among ImPrEP participants from Mexico	7
Supplementary Table 5. Factors associated with incident and recurrent bacterial STI among ImPrEP participants from Peru	9
ImPrEP Study Group	11
ImPrEP Study Sites	13

Supplementary Table 1. Baseline characteristics of participants according to inclusion in the prevalent STI analysis (per country)

Characteristics	Brazil				Mexico				Peru			
	Total included in ImPrEP n (%) N=3928	Participants included in Prevalent STI analysis			Total included in ImPrEP n (%) N=3288	Participants included in prevalent STI analysis			Total included in ImPrEP n (%) N=2293	Participants included in prevalent STI analysis		
		Yes N=3640 (92.7%)	No N=288 (7.3%)	p-value[a]		Yes N=2671 (81.2%)	No N=617 (18.8%)	p-value[a]		Yes N=2214 (96.6%)	No N=79 (3.4%)	p-value[a]
**Gender**				0.30				0.019				0.014
Cisgender man	3733 (95.0)	3463 (95.1)	270 (93.8)		3193 (97.1)	2585 (96.8)	608 (98.5)		2040 (89.0)	1963 (88.7)	77 (97.5)	
Transgender woman	195 (5.0)	177 (4.9)	18 (6.3)		95 (2.9)	86 (3.2)	9 (1.5)		253 (11.0)	251 (11.3)	2 (2.5)	
**Age group (years)**												
Median (IQR)	3928 (100.0)	27 (23-34)	29 (24-35)	<0.0001	3288 (100.0)	30 (26-36)	30 (25-35)	0.020	2293 (100.0)	28 (25-33)	27 (23-33)	0.11
18-24	1033 (26.3)	924 (25.4)	109 (37.8)	<0.0001	643 (19.6)	538 (20.1)	105 (17.0)	0.18	805 (35.1)	787 (35.5)	18 (22.8)	0.029
25-30	1216 (31.0)	1136 (31.2)	80 (27.8)		1156 (35.2)	938 (35.1)	218 (35.3)		695 (30.3)	662 (29.9)	33 (41.8)	
>30	1679 (42.7)	1580 (43.4)	99 (34.4)		1489 (45.3)	1195 (44.7)	294 (47.6)		793 (34.6)	765 (34.6)	28 (35.4)	
**Race**				0.0004				0.092				0.71
White	1868 (47.6)	1760 (48.4)	108 (37.5)		453 (13.8)	355 (13.3)	98 (15.9)		233 (10.2)	224 (10.1)	9 (11.4)	
Non-white	2060 (52.4)	1880 (51.6)	180 (62.5)		2835 (86.2)	2316 (86.7)	519 (84.1)		2060 (89.8)	1990 (89.9)	70 (88.6)	
**Education**				0.015[b]				0.49[b]				0.0029[b]
Primary	52 (1.3)	45 (1.2)	7 (2.4)		23 (0.7)	19 (0.7)	4 (0.6)		38 (1.7)	38 (1.7)	0 (0.0)	
Secondary	775 (19.7)	704 (19.3)	71 (24.7)		182 (5.5)	154 (5.8)	28 (4.5)		705 (30.7)	693 (31.3)	12 (15.2)	
Post-secondary	3101 (78.9)	2891 (79.4)	210 (72.9)		3083 (93.8)	2498 (93.5)	585 (94.8)		1550 (67.6)	1483 (67.0)	67 (84.8)	
**Main reason to attend the service**				0.82				0.13				0.0008
Seeking PrEP	3774 (96.1)	3498 (96.1)	276 (95.8)		3126 (95.1)	2532 (94.8)	594 (96.3)		1448 (63.1)	1384 (62.5)	64 (81.0)	
Other	154 (3.9)	142 (3.9)	12 (4.2)		162 (4.9)	139 (5.2)	23 (3.7)		845 (36.9)	830 (37.5)	15 (19.0)	
**Previous PEP use**				0.17				0.78				0.48[b]
Yes	1119 (28.5)	1047 (28.8)	72 (25.0)		510 (15.5)	412 (15.4)	98 (15.9)		63 (2.7)	60 (2.7)	3 (3.8)	
No	2809 (71.5)	2593 (71.2)	216 (75.0)		2778 (84.5)	2259 (84.6)	519 (84.1)		2230 (97.3)	2154 (97.3)	76 (96.2)	

**Number of sex partners**												
Median (IQR)	3928 (100.0)	7 (2-25)	5 (2-15)	0.0005	3288 (100.0)	7 (3-15)	8 (4-15)	0.30	2293 (100.0)	5 (2-11)	4 (2-10)	0.90
0-1	766 (19.5)	710 (19.5)	56 (19.4)	0.087	287 (8.7)	216 (8.1)	71 (11.5)	0.023	345 (15.0)	328 (14.8)	17 (21.5)	0.15
2-3	776 (19.8)	733 (20.1)	43 (14.9)		505 (15.4)	410 (15.4)	95 (15.4)		625 (27.3)	609 (27.5)	16 (20.3)	
4+	2386 (60.7)	2197 (60.4)	189 (65.6)		2496 (75.9)	2045 (76.6)	451 (73.1)		1323 (57.7)	1277 (57.7)	46 (58.2)	
**Receptive CAS**				0.034				0.14				0.043
Yes	2557 (65.1)	2353 (64.6)	204 (70.8)		2340 (71.2)	1916 (71.7)	424 (68.7)		1355 (59.1)	1317 (59.5)	38 (48.1)	
No	1371 (34.9)	1287 (35.4)	84 (29.2)		948 (28.8)	755 (28.3)	193 (31.3)		938 (40.9)	897 (40.5)	41 (51.9)	
**CAS with partners living with HIV**				0.0014				0.0084				0.0019
Yes	824 (21.0)	765 (21.0)	59 (20.5)		831 (25.3)	648 (24.3)	183 (29.7)		237 (10.3)	221 (10.0)	16 (20.3)	
No	1144 (29.1)	1085 (29.8)	59 (20.5)		752 (22.9)	607 (22.7)	145 (23.5)		744 (32.4)	729 (32.9)	15 (19.0)	
Don't know	1960 (49.9)	1790 (49.2)	170 (59.0)		1704 (51.8)	1415 (53.0)	289 (46.8)		1312 (57.2)	1264 (57.1)	48 (60.8)	
**Transactional sex**				0.23				0.0033				0.19
Yes	397 (10.1)	362 (9.9)	35 (12.2)		732 (22.3)	622 (23.3)	110 (17.8)		514 (22.4)	501 (22.6)	13 (16.5)	
No	3531 (89.9)	3278 (90.1)	253 (87.8)		2555 (77.7)	2048 (76.7)	507 (82.2)		1779 (77.6)	1713 (77.4)	66 (83.5)	
**Binge drinking**				0.13				0.021				0.077
Yes	2566 (65.3)	2366 (65.0)	200 (69.4)		2009 (61.1)	1657 (62.1)	352 (57.1)		1679 (73.2)	1628 (73.5)	51 (64.6)	
No	1362 (34.7)	1274 (35.0)	88 (30.6)		1278 (38.9)	1013 (37.9)	265 (42.9)		614 (26.8)	586 (26.5)	28 (35.4)	
**Stimulant use**				0.13				0.14				0.81[b]
Yes	715 (18.2)	672 (18.5)	43 (14.9)		888 (27.0)	736 (27.6)	152 (24.6)		140 (6.1)	135 (6.1)	5 (6.3)	
No	3213 (81.8)	2968 (81.5)	245 (85.1)		2400 (73.0)	1935 (72.4)	465 (75.4)		2153 (93.9)	2079 (93.9)	74 (93.7)	
**Poppers use**				0.56				0.60				0.19[b]
Yes	279 (7.1)	261 (7.2)	18 (6.3)		1539 (46.8)	1256 (47.0)	283 (45.9)		121 (5.3)	114 (5.1)	7 (8.9)	
No	3649 (92.9)	3379 (92.8)	270 (93.8)		1749 (53.2)	1415 (53.0)	334 (54.1)		2172 (94.7)	2100 (94.9)	72 (91.1)	

[a]Chi-squared test, [b]Fisher's exact test

Supplementary Table 2. Baseline characteristics of participants according to inclusion in the incidence and recurrent STI analyses (per country)

Characteristics	Brazil				Mexico				Peru			
	Total included in ImPrEP n (%) N=3928	Participants included in the incident and reccurent STI analyses			Total included in ImPrEP n (%) N=3288	Participants included in the incident and reccurent STI analyses			Total included in ImPrEP n (%) N=2293	Participants included in the incident and reccurent STI analyses		
		Yes N=3447 (87.8%)	No N=481 (12.2%)	p-value		Yes N=2486 (75.6%)	No N=802 (24.4%)	p-value		Yes N=1625 (70.9%)	No N=668 (29.1%)	p-value
**Gender**				<0.0001				<0.0001				0.0046
Cisgender man	3733 (95.0)	3309 (96.0)	424 (88.1)		3193 (97.1)	2437 (98.0)	756 (94.3)		2040 (89.0)	1465 (90.2)	575 (86.1)	
Transgender woman	195 (5.0)	138 (4.0)	57 (11.9)		95 (2.9)	49 (2.0)	46 (5.7)		253 (11.0)	160 (9.8)	93 (13.9)	
**Age group (years)**												
Median (IQR)	3928 (100.0)	27 (23-32)	29 (25-36)	<0.0001	3288 (100.0)	29 (24-33)	30 (26-35)	<0.0001	2293 (100.0)	26 (22-32)	27 (23-34)	<0.0001
18-24	1033 (26.3)	854 (24.8)	179 (37.2)	<0.0001	643 (19.6)	435 (17.5)	208 (25.9)	<0.0001	805 (35.1)	542 (33.4)	263 (39.4)	0.0014
25-30	1216 (31.0)	1055 (30.6)	161 (33.5)		1156 (35.2)	859 (34.6)	297 (37.0)		695 (30.3)	485 (29.8)	210 (31.4)	
>30	1679 (42.7)	1538 (44.6)	141 (29.3)		1489 (45.3)	1192 (47.9)	297 (37.0)		793 (34.6)	598 (36.8)	195 (29.2)	
**Race**				<0.0001				0.14				0.53
White	1868 (47.6)	1685 (48.9)	183 (38.0)		453 (13.8)	355 (14.3)	98 (12.2)		233 (10.2)	161 (9.9)	72 (10.8)	
Non-white	2060 (52.4)	1762 (51.1)	298 (62.0)		2835 (86.2)	2131 (85.7)	704 (87.8)		2060 (89.8)	1464 (90.1)	596 (89.2)	
**Education**				<0.0001				<0.0001				<0.0001
Primary	52 (1.3)	36 (1.0)	16 (3.3)		23 (0.7)	17 (0.7)	6 (0.7)		38 (1.7)	23 (1.4)	15 (2.2)	
Secondary	775 (19.7)	640 (18.6)	135 (28.1)		182 (5.5)	112 (4.5)	70 (8.7)		705 (30.7)	439 (27.0)	266 (39.8)	
Post-secondary	3101 (78.9)	2771 (80.4)	330 (68.6)		3083 (93.8)	2357 (94.8)	726 (90.5)		1550 (67.6)	1163 (71.6)	387 (57.9)	
**Main reason to attend the service**				0.0052				0.93				<0.0001
Seeking PrEP	3774 (96.1)	3323 (96.4)	451 (93.8)		3126 (95.1)	2364 (95.1)	762 (95.0)		1448 (63.1)	1084 (66.7)	364 (54.5)	
Other	154 (3.9)	124 (3.6)	30 (6.2)		162 (4.9)	122 (4.9)	40 (5.0)		845 (36.9)	541 (33.3)	304 (45.5)	
**Previous PEP use**				0.0001				0.0014				0.039
Yes	1119 (28.5)	1018 (29.5)	101 (21.0)		510 (15.5)	414 (16.7)	96 (12.0)		63 (2.7)	52 (3.2)	11 (1.6)	
No	2809 (71.5)	2429 (70.5)	380 (79.0)		2778 (84.5)	2072 (83.3)	706 (88.0)		2230 (97.3)	1573 (96.8)	657 (98.4)	

**Number of sex partners**												
Median (IQR)	3928 (100.0)	4 (2, 12)	5 (2-15)	0.17	3288 (100.0)	7 (3-15)	8 (4-15)	0.19	2293 (100.0)	4 (2-10)	5 (2-10)	0.096
0-1	766 (19.5)	667 (19.4)	99 (20.6)	0.13	287 (8.7)	220 (8.8)	67 (8.4)	0.082	345 (15.0)	237 (14.6)	108 (16.2)	0.092
2-3	776 (19.8)	667 (19.4)	109 (22.7)		505 (15.4)	362 (14.6)	143 (17.8)		625 (27.3)	427 (26.3)	198 (29.6)	
4+	2386 (60.7)	2113 (61.3)	273 (56.8)		2496 (75.9)	1904 (76.6)	592 (73.8)		1323 (57.7)	961 (59.1)	362 (54.2)	
**Receptive CAS**				0.081				0.70				0.097
Yes	2557 (65.1)	2261 (65.6)	296 (61.5)		2340 (71.2)	1765 (71.0)	575 (71.7)		1355 (59.1)	978 (60.2)	377 (56.4)	
No	1371 (34.9)	1186 (34.4)	185 (38.5)		948 (28.8)	721 (29.0)	227 (28.3)		938 (40.9)	647 (39.8)	291 (43.6)	
**CAS with partners living with HIV***				0.032				0.0014				0.0080
Yes	824 (21.0)	739 (21.4)	85 (17.7)		831 (25.3)	667 (26.8)	164 (20.5)		237 (10.3)	187 (11.5)	50 (7.5)	
No	1144 (29.1)	1014 (29.4)	130 (27.0)		752 (22.9)	561 (22.6)	191 (23.8)		744 (32.4)	532 (32.7)	212 (31.7)	
Don't know	1960 (49.9)	1694 (49.1)	266 (55.3)		1704 (51.8)	1258 (50.6)	446 (55.7)		1312 (57.2)	906 (55.8)	406 (60.8)	
**Transactional sex***				<0.0001				0.0007				<0.0001
Yes	397 (10.1)	315 (9.1)	82 (17.0)		732 (22.3)	519 (20.9)	213 (26.6)		514 (22.4)	334 (20.6)	180 (26.9)	
No	3531 (89.9)	3132 (90.9)	399 (83.0)		2555 (77.7)	1967 (79.1)	588 (73.4)		1779 (77.6)	1291 (79.4)	488 (73.1)	
**Binge drinking***				0.32				0.78				0.064
Yes	2566 (65.3)	2242 (65.0)	324 (67.4)		2009 (61.1)	1516 (61.0)	493 (61.5)		1679 (73.2)	1172 (72.1)	507 (75.9)	
No	1362 (34.7)	1205 (35.0)	157 (32.6)		1278 (38.9)	970 (39.0)	308 (38.5)		614 (26.8)	453 (27.9)	161 (24.1)	
**Stimulant use**				0.23				0.74				0.42
Yes	715 (18.2)	618 (17.9)	97 (20.2)		888 (27.0)	675 (27.2)	213 (26.6)		140 (6.1)	95 (5.8)	45 (6.7)	
No	3213 (81.8)	2829 (82.1)	384 (79.8)		2400 (73.0)	1811 (72.8)	589 (73.4)		2153 (93.9)	1530 (94.2)	623 (93.3)	
**Poppers use**				0.33				0.0002				0.0008
Yes	279 (7.1)	250 (7.3)	29 (6.0)		1539 (46.8)	1209 (48.6)	330 (41.1)		121 (5.3)	102 (6.3)	19 (2.8)	
No	3649 (92.9)	3197 (92.7)	452 (94.0)		1749 (53.2)	1277 (51.4)	472 (58.9)		2172 (94.7)	1523 (93.7)	649 (97.2)	
**Bacterial IST at baseline**				0.32†				0.75†				0.88†
Yes	911 (23.2)	812 (23.6)	99 (20.6)		655 (19.9)	481 (19.3)	174 (21.7)		618 (27.0)	440 (27.1)	178 (26.6)	
No	2729 (69.5)	2399 (69.6)	330 (68.6)		2016 (61.3)	1493 (60.1)	523 (65.2)		1596 (69.6)	1131 (69.6)	465 (69.6)	
Not tested	288 (7.3)	236 (6.8)	52 (10.8)		617 (18.8)	512 (20.6)	105 (13.1)		79 (3.4)	54 (3.3)	25 (3.7)	

* Dentotes there is one missing case across any of the variables, †Chi-squared test calculated using only the information from performed tests.

Supplementary Table 3. Factors associated with incident and recurrent bacterial STI diagnoses among ImPrEP participants from Brazil

	Incident bacterial STI				Recurrent Bacterial STI			
	Univariable analyses		Multivariable analysis		Univariable analyses		Multivariable analysis	
	HR (95%CI)	p-value	aHR (95%CI)	p-value	HR (95%CI)	p-value	aHR (95%CI)	p-value
**Gender**								
Cisgender man	1		-	-	1	-	-	-
Transgender woman	1.16 (0.87-1.55)	0.30	-	-	0.66 (0.36-1.19)	0.17	-	-
**Age (years)**								
18-24	1.26 (1.10-1.45)	0.0013	1.19 (1.03-1.38)	0.019	1.64 (1.32-2.03)	<0.0001	1.52 (1.22-1.90)	0.0002
25-30	1.12 (0.99-1.28)	0.083	1.07 (0.93-1.22)	0.33	1.24 (1.00-1.52)	0.047	1.16 (0.94-1.43)	0.17
>30	1	-	1.00	-	1	-	1	-
**Race**								
White	1	-	1.00	-	1	-	1	-
Non-white	1.12 (1.00-1.25)	0.053	1.10 (0.98-1.24)	0.089	1.19 (1.00-1.42)	0.057	1.12 (0.93-1.35)	0.22
**Education**								
Primary	1.10 (0.64-1.90)	0.73	-	-	1.08 (0.45-2.61)	0.87	-	-
Secondary	1.11 (0.96-1.28)	0.17	-	-	1.06 (0.83-1.34)	0.65	-	-
More than secondary	1	-	-	-	1	-	-	-
**Main reason to attend the service**								
Seeking PrEP	1	-	-	-	1	-	-	-
Other	1.09 (0.81-1.48)	0.56	-	-	1.16 (0.72-1.85)	0.54	-	-
**PEP use**								
Yes	1.26 (1.12-1.42)	0.0001	1.22 (1.08-1.37)	0.0010	1.26 (1.05-1.52)	0.013	1.17 (0.97-1.41)	0.095
No	1	-	1.00	-	1	-	1	-
**Number of sex partners**[1]								
0-1	1	-	1.00	-	1	-	1	-
2-3	1.60 (1.33-1.92)	<0.0001	1.56 (1.29-1.87)	<0.0001	2.12 (1.56-2.88)	<0.0001	1.99 (1.46-2.71)	<0.0001
4+	2.33 (2.01-2.71)	<0.0001	2.09 (1.78-2.45)	<0.0001	3.13 (2.41-4.06)	<0.0001	2.60 (1.99-3.39)	<0.0001
**Receptive CAS**[2]								
Yes	1.80 (1.60-2.03)	<0.0001	1.60 (1.41-1.80)	<0.0001	2.30 (1.88-2.81)	<0.0001	1.94 (1.58-2.38)	<0.0001
No	1	-	1	-	1	-	1	-
**CAS with partner(s) with HIV**[2]								

Yes	0.84 (0.71-0.99)	0.038	0.88 (0.74-1.04)	0.12	0.82 (0.63-1.08)	0.16	-	-
No	1	-	1	-	1	-	-	-
I don't know	1.03 (0.90-1.17)	0.66	0.87 (0.76-1.00)	0.051	1.05 (0.85-1.29)	0.65	-	-
**Transactional sex**[2]								
Yes	1.28 (1.07-1.54)	0.0067	0.94 (0.78-1.13)	0.45	1.60 (1.23-2.08)	0.0004	1.05 (0.80-1.39)	0.70
No	1		1	-	1		1	-
**Binge drinking**[2]								
Yes	1.09 (0.98-1.22)	0.12	-	-	1.10 (0.92-1.32)	0.28	-	-
No	1.00	-	-	-	1	-	-	-
**Stimulant use**[2]								
Yes	1.26 (1.09-1.47)	0.0023	0.99 (0.85-1.16)	0.94	1.43 (1.14-1.80)	0.0023	1.07 (0.84-1.37)	0.57
No	1.00	-	1	-	1.00	-	1	-
**Poppers**[2]								
Yes	1.51 (1.20-1.90)	0.0004	1.22 (0.96-1.55)	0.11	1.52 (1.06-2.18)	0.022	1.13 (0.78-1.65)	0.51
No	1.00	-		-	1.00	-	1	-
**Bacterial STI at baseline**								
Yes	1.98 (1.75-2.24)	<0.0001	1.83 (1.62-2.08)	<0.0001	2.27 (1.89-2.73)	<0.0001	2.01 (1.67-2.41)	<0.0001
No	1.00	-	1					

[1] In the last 3 months; [2] In the last 6 months

Supplementary Table 4. Factors associated with incident and recurrent bacterial STI among ImPrEP participants from Mexico

	**Incident bacterial STI**				**Recurrent Bacterial STI**			
	**Univariable analyses**		**Multivariable analysis**		**Univariable analyses**		**Multivariable analysis**	
	HR (95%CI)	p-value	aHR (95%CI)	p-value	HR (95%CI)	p-value	aHR (95%CI)	p-value
**Gender**								
Cisgender man	1		-	-	1	-	-	-
Transgender woman	0.92 (0.50-1.66)	0.77	-	-	1.20 (0.49-2.92)	0.68	-	-
**Age (years)**								
18-24	1.30 (1.08-1.58)	0.0062	1.26 (1.04-1.53)	0.020	1.32 (0.98-1.78)	0.068	1.28 (0.94-1.74)	0.11
25-30	1.12 (0.96-1.31)	0.16	1.09 (0.93-1.28)	0.29	1.15 (0.90-1.48)	0.25	1.12 (0.88-1.45)	0.36
>30	1	-	1	-	1	-	1	-
**Race**								
White	1	-	1	-	1	-	-	-
Non-white	1.43 (1.15-1.79)	0.0015	1.41 (1.13-1.77)	0.0024	1.27 (0.91-1.78)	0.16	-	-
**Education**								
Primary (complete or incomplete)	0.84 (0.35-2.03)	0.70	-	-	0.99 (0.25-4.01)	0.99	1.16 (0.28-4.75)	0.84
Secondary (complete or incomplete)	1.20 (0.86-1.67)	0.28	-	-	1.91 (1.22-2.97)	0.0046	1.77 (1.12-2.79)	0.014
More than secondary	1	-	-	-	1	-	1	-
**Main reason to attend the service**								
Seeking PrEP	1	-	-	-	1	-	-	-
Other	1.17 (0.84-1.62)	0.35	-	-	1.26 (0.75-2.13)	0.38	-	
**PEP use**								
Yes	0.96 (0.80-1.15)	0.63	-	-	0.85 (0.63-1.15)	0.28	-	-
No	1	-	-	-	1	-		
**Number of sex partners**[1]								
0-1	1	-	1	-	1	-	1	-
2-3	1.18 (0.91-1.52)	0.22	1.15 (0.89-1.49)	0.29	1.30 (0.83-2.04)	0.25	1.24 (0.79-1.95)	0.35
4+	1.32 (1.06-1.63)	0.012	1.17 (0.94-1.46)	0.17	1.69 (1.16-2.46)	0.0064	1.42 (0.96-2.08)	0.077
**Receptive CAS**[2]								
Yes	1.00 (0.73-1.37)	0.99	-	-	1.26 (0.85-1.88)	0.25	-	-
No	1	-	-	-	1	-	-	-

**CAS with partner(s) with HIV[2]**								
Yes	1.31 (1.07-1.60)	0.0079	1.31 (1.07-1.60)	0.0093	1.33 (0.96-1.84)	0.082	1.32 (0.95-1.83)	0.092
No	1	-	1	-	1	-	1	-
I don't know	1.31 (1.09-1.58)	0.0037	1.30 (1.08-1.56)	0.0063	1.50 (1.11-2.01)		1.45 (1.08-1.96)	0.014
**Transactional sex[2]**								
Yes	1.26 (1.06-1.49)	0.0081	1.10 (0.92-1.31)	0.31	1.03 (0.78-1.36)	0.84	-	-
No	1		1.00	-	1		-	-
**Binge drinking[2]**								
Yes	0.99 (0.86-1.14)	0.93	-	-	1.10 (0.88-1.37)	0.41	-	-
No	1.00	-	-	-	1	-	-	-
**Stimulant use[2]**								
Yes	1.24 (1.05-1.46)	0.011	1.12 (0.94-1.34)	0.20	1.17 (0.90-1.51)	0.25	-	-
No	1.00	-	1	-	1	-	-	-
**Poppers**								
Yes	1.32 (1.14-1.51)	0.0001	1.20 (1.03-1.39)	0.020	1.54 (1.24-1.92)	<0.0001	1.39 (1.10-1.74)	0.0052
No	1.00	-	1	-	1	-	1	-
**Bacterial STI at enrollment**								
Yes	1.59 (1.34-1.88)	<0.0001	1.52 (1.29-1.80)	<0.0001	1.92 (1.46-2.51)	<0.0001	1.80 (1.37-2.36)	<0.0001
No	1.00	-	1					

[1] In the last 3 months; [2] In the last 6 months

Supplementary Table 5. Factors associated with incident and recurrent bacterial STI among ImPrEP participants from Peru

	Incident bacterial STI				Recurrent Bacterial STI			
	Univariable analyses		Multivariable analysis		Univariable analyses		Multivariable analysis	
	HR (95%CI)	p-value	aHR (95%CI)	p-value	HR (95%CI)	p-value	aHR (95%CI)	p-value
**Gender**								
Cisgender man	1		1		1	-	1	-
Transgender woman	1.48 (1.11-1.96)	0.0071	1.19 (0.84-1.67)	0.33	1.59 (0.96-2.66)	0.073	1.44 (0.80-2.60)	0.23
**Age (years)**								
18-24	1.41 (1.10-1.81)	0.0071	1.41 (1.09-1.82)	0.0086	1.67 (1.03-2.70)	0.038	1.75 (1.07-2.88)	0.027
25-30	1.30 (1.00-1.70)	0.051	1.33 (1.01-1.73)	0.039	1.38 (0.81-2.34)	0.24	1.40 (0.82-2.40)	0.22
>30	1	-	1	-	1	-	1	-
**Race**								
White	1	-	-	-	1	-	-	-
Non-white	0.91 (0.65-1.28)	0.60	-	-	1.07 (0.53-2.14)	0.85	-	-
**Education**								
Primary	0.90 (0.29-2.81)	0.85	0.77 (0.24-2.47)	0.67	3.38 (0.81-14.10)	0.094	2.85 (0.65-12.60)	0.16
Secondary	1.26 (1.01-1.57)	0.039	1.06 (0.83-1.35)	0.64	1.25 (0.81-1.92)	0.30	0.97 (0.61-1.55)	0.89
More than secondary	1	-	1	-	1	-	1	-
**Main reason to attend the service**								
Seeking PrEP	1	-	-	-	1	-	-	-
Other	0.99 (0.79-1.22)	0.90	-	-	1.07 (0.71-1.62)	0.73	-	-
**PEP use**								
Yes	1.05 (0.57-1.91)	0.89	-	-	1.43 (0.52-3.96)	0.48	-	-
No	1	-	-	-	1	-	-	-
**Number of sex partners**[1]								
0-1	1	-	1	-	1	-	1	-
2-3	1.62 (1.16-2.25)	0.0047	1.51 (1.08-2.11)	0.016	2.56 (1.21-5.46)	0.015	2.48 (1.16-5.30)	0.019
4+	1.76 (1.30-2.39)	0.0003	1.57 (1.15-2.15)	0.0048	2.74 (1.34-5.58)	0.0060	2.53 (1.23-5.22)	0.013
**Receptive CAS**[2]								
Yes	1.58 (1.25-2.00)	0.0002	1.36 (1.06-1.74)	0.014	1.45 (0.91-2.30)	0.12	-	-
No	1	-	1	-	1	-	-	-

**CAS with partner(s) with HIV**[2]								
Yes	0.97 (0.68-1.39)	0.87	-	-	0.66 (0.30-1.43)	0.29	-	-
No	1	-	-	-	1	-	-	-
I don't know	1.05 (0.84-1.32)	0.67	-	-	1.05 (0.68-1.63)	0.81	-	-
**Transactional sex**[2]								
Yes	1.44 (1.14-1.82)	0.0023	1.18 (0.90-1.54)	0.24	1.27 (0.81-2.01)	0.30	-	-
No	1		1	-	1		-	-
**Binge drinking**[2]								
Yes	1.13 (0.91-1.41)	0.27	-	-	0.99 (0.66-1.50)	0.98	-	-
No	1	-	-	-	1	-	-	-
**Stimulant use**[2]								
Yes	1.13 (0.66-1.94)	0.65	-	-	3.61 (1.86-7.03)	0.0002	2.94 (1.50-5.79)	0.0021
No	1	-	-	-	1	-	1	-
**Poppers**								
Yes	1.44 (0.88-2.35)	0.14	-	-	1.40 (0.51-3.86)	0.51	-	-
No	1	-	-	-	1	-	-	-
**Bacterial STI at baseline**								
Yes	1.58 (1.27-1.96)	<0.0001	1.42 (1.14-1.77)	0.0021	2.41 (1.60-3.62)	<0.0001	2.13 (1.40-3.23)	0.0005
No	1	-	1		1		1	

[1] In the last 3 months; [2] In the last 6 months

ImPrEP Study Group

Brazil

Marcus Vinicius Lacerda, José Valdez Madruga, Alessandro Farias, Josué N Lima, Ronaldo Zonta, Lilian Lauria, J. David Urbaez-Brito, Polyana d'Albuquerque, Claudio Palombo, Paulo Ricardo de Alencastro, Raquel Keiko de Luca Ito, Julio Moreira, João L. de Benedetti, Fabio V. Maria, Paula M. Luz, Lucilene Freitas, Kim Geraldo, Monica Derrico, Sandro Nazer, Tania Kristic, Renato Girade (*in memoriam*), Renato Lima, Antônio R. de Carvalho, Carla Rocha, Pedro Leite, Marcio Lessa, Marilia Santini, Daniel R. B. Bezerra, Cleo de Oliveira Souza, Jacinto Corrêa, Marcelo Alves, Carolina Souza, Camilla Portugal, Mônica dos Santos Valões, Gabriel Lima Mota, Joyce Alves Gomes, Cynthia Ferreira Lima Falcão, Fernanda Falcão Riberson, Luciano Melo, Talita Andrade Oliva, Agnaldo Moreira de Oliveira Júnior, Bruna Fonseca, Leonor Henriette de Lannoy, Ludymilla Anderson Santiago Carlos, João Paulo da Cunha, Sonia Maria de Alencastro Coracini, Thiago Oliveira Rodrigues, Emília Regina Scharf Mettrau, Kelly Vieira Meira; Heder Tavares, Ana Paula Nunes Viveiros Valeiras, Taiane Miyake Alves de Carvalho Rocha, Alex Amorim, Patrícia Sabadini, Luiz Gustavo Córdoba; Caio Gusmão, Erika Faustino, Julia Soares da Silva Hansen, Agatha Mirian Cunha, Neuza Uchiyama Nishimura, Jaime Eduardo Flygare Razo Prereira dos Santos, Aline Barnabé Cano, Willyam Magnum Telles Dias, Magô Tonhon, Tania Regina Rezende, Alex Gomes, Eloá dos Santos Rodrigues, Maria das Dores Aires Carneiro, Alexandre Castilho, Mariana Carvalho.

Mexico

Sergio Bautista-Aredondo, Helee Vermandere, Steven Diaz, Dulce Diaz-Sosa, Centli Guillen Diaz-Barriga, Lorena Hernández, Rebeca Robles, Maria Elena Medina-Mora, Marcela González, Ivonne Huerta Icelo, Araczy Martinez Davalos, José Gomez Castro, Luis Obed Ocampo Valdez, Fernanda Ramírez Barajas, Verónica Ruiz González, Galileo Vargas Guadarrama, Israel Macías, Jehovani Tena Sánchez, Juan Pablo Osuna Noriega, H· Rodrigo Moheno M·, Jorge M· Bernal Ramírez, Víctor Dante Galicia Juarez, Gerardo Vizcaíno, Francisco Javier Arjona.

Peru

Cesar Vidal Osco Tamayo, Hector Javier Salvatierra Flores, Yovanna Margot Cabrera Santa Cruz, Ricardo Martín Moreno Aguayo, Gino Calvo, Silver Vargas, Oliver Elorreaga, Ximena

Gutierrez, Fernando Olivos, Damaris Caviedes, Daniella Adriazola, Eduardo Juárez, Gabriela Mariño, Jazmin Qquellon, Francesca Vasquez, Jean Pierre Jiron, Sonia Flores, Karen Campos.

ImPrEP Study Sites

Brazil

Fundação de Medicina Tropical (Manaus, Amazonas), Hospital Universitário Oswaldo Cruz (Recife, Pernambuco), CEDAP – Centro Estadual Especializado em Diagnóstico, Assistência e Pesquisa (Salvador, Bahia), Hospital Dia Asa Sul (Brasília, Distrito Federal), Instituto Nacional de Infectologia Evandro Chagas, Fundação Oswaldo Cruz INI-Fiocruz (Rio de Janeiro), Hospital Municipal Rocha Maia (Rio de Janeiro), Hospital Municipal Carlos Tortelly (Niterói, Rio de Janeiro), Centro de Referência em DST/AIDS- AMDA (Campinas, São Paulo), Centro de Referência e Treinamento em DST/AIDS – CRT-SP (São Paulo), SAE DST/AIDS – CECI (São Paulo), SAE DST/AIDS – Fidélis Ribeiro (São Paulo), SAE Adulto (Santos, São Paulo), Poli Centro (Florianópolis, Santa Catarina), SAT – Sanatório Partenon (Porto Alegre, Rio Grande do Sul).

Mexico

Clínica Especializada Condesa (Cuauhtémoc, Mexico City), Fundación Unidos por un México Vivo A.C. (Cuauhtémoc, Mexico City), Comité Humanitario de Esfuerzo Compartido Contra El Sida A.C. (Guadalajara, Jalisco), Solidaridad Ed Thomas A·C· (Puerto Vallarta, Jalisco).

Peru

Centro de Referencia de Infecciones de Transmisión Sexual del Centro Materno Infantil San José (Lima), Centro de Referencia de Infecciones de Transmisión Sexual del Centro Materno Infantil Tahuantinsuyo Bajo (Lima), Centro de Referencia de Infecciones de Transmisión Sexual del Centro de Salud Alberto Barton (Callao), Centro de Referencia de Infecciones de Transmisión Sexual de Caja de Agua (Lima), Centro de Referencia de Infecciones de Transmisión Sexual del Hospital Amazónico Pucallpa (Ucayali), Centro de Referencia de Infecciones de Transmisión Sexual del Hospital La Caleta Chimbote (Ancash), Centro de Referencia de Infecciones de Transmisión Sexual del Hospital Regional Ica (Ica), Centro de Referencia de Infecciones de Transmisión Sexual del Hospital Regional Trujillo (La Libertad), Investigaciones Médicas en Salud, INMENSA (Lima), Centro de Referencia de Infecciones de Transmisión Sexual del Hospital San Juan De Dios, Pisco (Ica).
